# Differential Stability of the Crystallographic Interfaces of Mu- and Kappa-Opioid Receptors

**DOI:** 10.1371/journal.pone.0090694

**Published:** 2014-02-28

**Authors:** Jennifer M. Johnston, Marta Filizola

**Affiliations:** Department of Structural and Chemical Biology, Icahn School of Medicine at Mount Sinai, New York, New York, United States of America; German Research School for Simulation Science, Germany

## Abstract

The recent mu-opioid receptor (MOPr) and kappa-opioid receptor (KOPr) crystal structures have inspired hypotheses of physiologically relevant dimerization contacts, specifically: a closely packed interface involving transmembrane (TM) helices TM5 and TM6, and a less compact interface, involving TM1, TM2, and helix 8 (H8). While the former was only found in MOPr crystals, similar arrangements of the latter were identified for both KOPr and MOPr. The relevance of these interfaces outside of a crystal lattice is called into question by the possibility that they might be influenced by the specific crystallization conditions. In this study, we have employed umbrella sampling molecular dynamics simulations of coarse-grained representations of the interacting MOPr or KOPr crystallographic structures, in the absence of the T4 lysozyme, and in an explicit lipid-water environment, to determine the strength of receptor dimerization at the different crystallographic interfaces. We note that the shape of the interface plays a dominant role in the strength of the interaction, and the pattern of contacting residues defines the shape of the potential of mean force. This information can be used to guide experiments aimed at exploring the role of dimerization in opioid receptor function.

## Introduction

Following several breakthroughs in crystallization techniques for G Protein-Coupled Receptors (GPCRs), particularly T4 lysozyme (T4L)-stabilized constructs and lipid cubic phase environments [1]–[3], several GPCR crystallographic structures have been solved in recent years, including those of opioid receptors [4]–[7]. While the majority of currently available crystal structures of GPCRs display single protomers in the asymmetric unit of the crystal, some of them, including those of the mu-opioid receptor (MOPr) and the kappa-opioid receptor (KOPr), have revealed parallel arrangements of interacting receptors. It is tempting to speculate that these parallel arrangements correspond to physiologically relevant dimeric configurations, notwithstanding the possibility that experimental conditions for crystallogenesis might have altered the receptor-receptor interactions that occur naturally in a living cell.

The two interfaces revealed by the MOPr crystals are i) a tightly packed interface involving transmembrane (TM) helices TM5 and TM6 from both interacting protomers (henceforth called the TM5,6/TM5,6 interface), and ii) a more loosely packed arrangement, with dominant symmetric contacts between TM1 and H8 in the adjacent protomers, as well as contacts between TM1 on one protomer, and TM2 on the adjacent protomer (henceforth called the TM1,2,H8/TM1,2,H8 interface). A similar, albeit not identical, TM5,6/TM5,6 interface has also been seen in five independent crystal structures of the chemokine receptor CXCR4 [8], which is another member of the same peptide subfamily of GPCRs to which opioid receptors belong. To the best of our knowledge, the physiological relevance of this putative dimeric interface simultaneously involving TM5 and TM6 has yet to be demonstrated *in vivo*. On the other hand, evidence exists from both biophysical and biochemical (mostly cross-linking) experiments that the TM5 helix may be part of a physiologically relevant, dimer interface for several GPCRs, including the MOPr cognate system, DOPr [9]. However, these interfaces typically involve TM4 and the intracellular loop 2 (IL2), in addition to TM5 (e.g., see [10], [11]).

The only interface present in the KOPr crystal structure is one involving interactions between TM1, TM2 and H8. This interface is similar, but not identical to the TM1,2,H8/TM1,2,H8 interface in the MOPr crystal. Unlike the TM5,6/TM5,6 interface, there are several recent experiments that support the participation of a TM1,2,H8/TM1,2,H8 interface in the dimerization of various GPCRs (e.g., see [11]–[13]). Most directly relevant to the opioid receptor field are recent experiments i) suggesting disruption of the proposed dimerization between MOPr and DOPr *in vivo* by an interfering peptide containing TM1 of MOPr [14], or ii) supporting the role of the carboxyl tail of the DOPr, as well as the IL3 of both MOPr and DOPr, in their heteromerization [15]. Taken together with all other available data, this information suggests that no clear consensus has emerged yet regarding relevant interfaces for opioid receptors in the dimerization process.

Being able to discriminate between dimer interfaces occurring naturally *in vivo* and possible artifacts generated by experimental conditions is extremely important, given the implications of dimerization on receptor function. A recent analysis [16] applying the Evolutionary Protein-Protein Interface Classifier to distinguish biological interfaces from some of the recently characterized crystallographic interfaces of family A GPCRs, including KOPr, concluded that, with the exception of the class F Smoothened receptor, none of the analyzed crystallographic interfaces of GPCRs exhibit the signature geometrical packing and evolution patterns expected for high-affinity, physiologically-relevant interfaces. While this is interesting, the results of an evolutionary analysis are limited by the number of relatively close sequence homologs that are available for the alignment, the uniformity of the distribution of identities in the homologs, and the simplicity of a sequence-based approach, which takes into account neither the energetics underlying the dimerization process, nor the role of the environment.

In this study, we sought to investigate both the viability and relative stability of the crystallographic interfaces identified for the MOPr and the KOPr in an explicit lipid-water environment, and in the absence of the T4L, in an effort to start prioritizing mutagenesis experiments aimed at exploring the role of dimerization in opioid receptor function.

## Materials and Methods

### System Setup

Missing loop segments from the crystallographic structures of the mouse MOPr (PDB ID: 4DKL) [5] (residues 263–270) and chain A of the human KOPr (PDB ID: 4DJH) [6] (residues 262 and 301–307) were built using ROSETTA version 2.2 [17]. The conformation for IL3 was selected as the minimum energy structure reported by ROSETTA. For MOPr, the lowest energy conformation that also did not interfere (inter-lock) with the IL3 of the adjacent receptor at the crystallographic TM5,6/TM5,6 interface was selected. Throughout this article we use the Ballesteros-Weinstein numbering scheme [18] to facilitate comparison between the receptor subtype TM regions. Accordingly, the first number of this scheme indicates the TM helix in which the residue in question resides, and the second number indicates the position of the residue relative to the most conserved residue in the helix, which is always numbered 50 (e.g., 1.45 indicates a residue 5 positions upstream from the most conserved residue in TM1).

**Figure 1:**
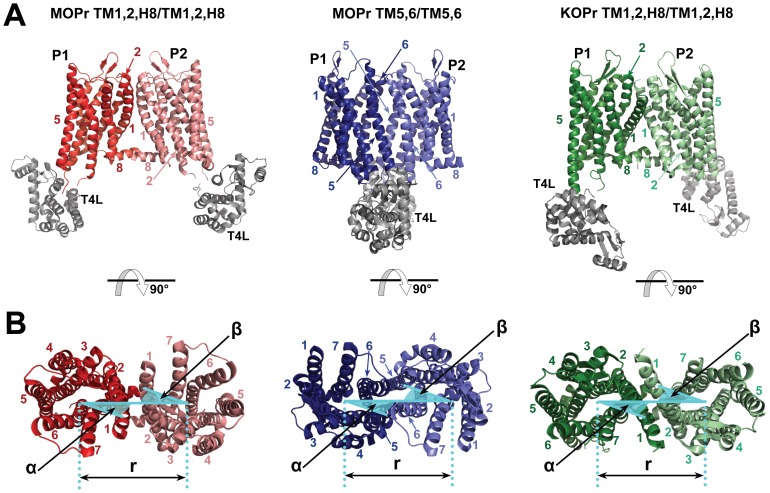
The crystallographic structures of the opioid receptors indicate putative interfacial interactions. A) Side view of crystallographic dimers. MOPr protomers interacting at a TM1,2,H8/TM1,2,H8 interface colored in red and pink from PDB ID: 4DKL. MOPr protomers interacting at a TM5,6/TM5,6 interface in blue and light blue, also from PDB ID 4DKL, and KOPr protomers interacting at a TM1,2,H8/TM1,2,H8 interface are shown in green and light green, from PDB ID: 4DJH. T4L is shown in gray in all cases. B) Extracellular view of crystallographic dimers, colored as above. Cyan overlay shows collective variables used to define the interface between protomer 1 and protomer 2 (P1 and P2). The PMF is calculated as a function of the separation *(r)* between the COMs of the TM regions of the two protomers. The orientation of the interface was maintained during the simulations by two harmonic restraints centered on the values of the angles, α and β. The angle α is the projection on the x, y plane of the angle calculated between the COM of the helix defining the interface, the COM of the helical bundle of the protomer bearing this helix (P1) and the COM of the adjacent protomer (P2). Angle β is the equivalent angle for the adjacent protomer.

The receptors, without N- and C-termini or ligands, were converted to a coarse-grained (CG) representation under the MARTINI force field (version 2.1) [19]–[21] and a modified elastic network was applied, as we have described, in detail, in earlier publications [22]. Unlike other GPCRs, which exhibit palmitoylated cysteines at the C-terminus, mutagenesis experiments [23], [24] have demonstrated that the only relevant site for palmitoylation in MOPr is at a specific cysteine position in the cytoplasmic side of TM3. Thus, receptors were palmitoylated at the C3.55 position, with a coarse-grained palmitoyl chain consisting of 4 C1-type beads, with a bond length of 0.47 nm, a force constant of 1250 kJ mol^−1^ nm^−2^ and angles of 180°, with a force constant 25 kJ mol^−1^. The ligand-free, CG systems were aligned onto the pairs of receptors associated along their crystallographic two-fold axes through two different interfaces involving TM1, TM2, and H8 or TM5 and TM6 in the case of MOPr, and one interface involving TM1, TM2, and H8 in the case of KOPr (Figure 1). These associated receptors were embedded into an explicit, MARTINI CG 1-palmitoyl,2-oleoyl-sn-glycero-3-phosphocholine (POPC)/10% cholesterol membrane, solvated with the MARTINI CG water model, and neutralizing counterions were added for each system. The resulting systems were minimized and equilibrated with harmonic restraints on protein backbone beads, of decreasing strength over 10 ns.

### Umbrella Sampling Simulations

Umbrella sampling simulations were carried out for the MOPr TM1,2,H8/TM1,2,H8, MOPr TM5,6/TM5,6, and KOPr TM1,2,H8/TM1,2,H8 systems, separately. The starting configurations for each umbrella sampling window simulation were taken from an unconverged metadynamics simulation in the same manner as we previously reported in the literature [25]. These starting configurations of ∼15,000 beads (the absolute value varies slightly between interface systems, but not between windows) were taken at varying values of separation (*r*) of the protomers, with spacing between windows of 0.04 nm, and the total number of windows required for complete sampling varied between 76 and 92 for the different systems. The receptors were separated along the line connecting the centers of mass (COMs) of their TM regions by adding a Gaussian bias. The orientation of each crystallographic interface was maintained during the separation by two harmonic restraints centered on the values of the angles, α and β, as defined in the crystal structures and illustrated in panel B of Figure 1. After minimization, 800 ns of simulation were performed with a third harmonic force restraining the value of *r* between the COM of the two protomers to that from the starting frame, and with a time step of 20 fs. Periodic boundary conditions were employed, and neighbor searching was performed every 10 steps. The Shift algorithm was used for electrostatic interactions with a cut-off of 1.2 nm. A single cut-off of 1.2 nm was used for Van der Waals interactions. Temperature and pressure coupling used the V-rescale and the Berendsen algorithms respectively. The range of *r* explored, and the orientation for the different interfaces and receptors are outlined in Table 1. All simulations were carried out using Gromacs version 4.0.5 [26], enhanced with the Plumed plugin [27].

**Table 1:** Key results and methodological aspects of PMF curves in Figure 2.

Receptor	Interface	Minimum*r* (nm)	F(*r*_min_)(kcal/mol)	K_Δ_ (µm^2^)	ΔG(kcal/mol)	Range *r* (nm)	α (rad)	β (rad)
MOPr	TM5,6/TM5,6	3.12	−19	4.4e3	−13	3.00–6.72	0.066–0.146	0.066–0.146
MOPr	TM1,2,H8/TM1,2,H8	3.80, 4.00	−12	7.6e-2	−7	3.72–6.72	0.04–0.12	0.11–0.19
KOPr	TM1,2,H8/TM1,2,H8	3.80	−44	4.6e22	−39	3.60–6.72	0.066–0.146	0.066–0.146

### Analyses of Simulations

**Figure 2:**
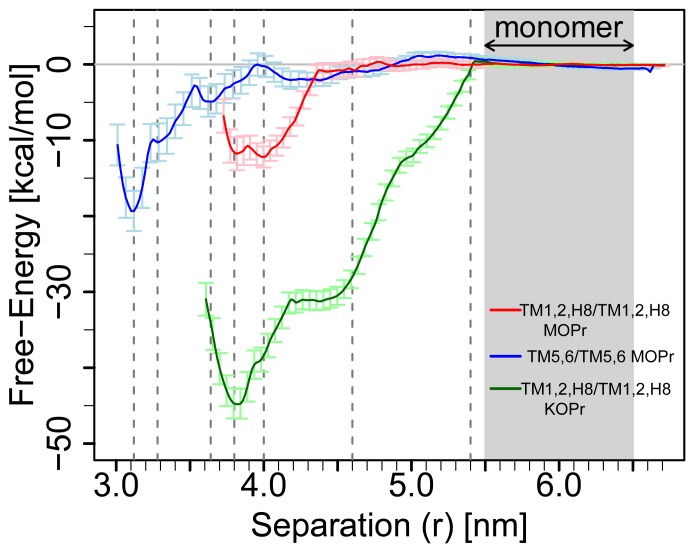
PMFs calculated from the umbrella sampling, coarse-grained simulations for each of the different interfaces: MOPr TM1,2,H8/TM1,2,H8 is shown in red (error bars in pink), MOPr TM5,6/TM5,6 is shown in blue (error bars in light blue), and KOPr TM1,2,H8/TM1,2,H8 is in green (error bars in light green). The grayed region denoted ‘monomer’ is that in which the PMF curves were aligned to free-energy = 0. The dashed lines, shown at r = 3.12 nm, 3.64 nm, 3.80 nm, 4.00 nm, 4.60 nm, and 5.40 nm, indicate the values of separation at which the inter-protomer contacts were assessed.

From the simulations in each window the value of *r* during the last 750 ns of simulation was collected. To remove the influence of the harmonic restraints on each window, each data point was reweighted by the sum of the exponential of the harmonic restraints applied to both α and β at that point. The reweighted data were then combined using the weighted histogram analysis method (WHAM) [28], [29] through an established, web-accessible implementation (http://membrane.urmc.rochester.edu/content/wham-releases) to calculate the potential of mean force (PMF) as a function of the separation between the COMs of the TM regions of interacting receptors. We have compared the PMFs from different interfaces by aligning the plateaus of the curves to zero; the plateaus represent the values of *r* at which the receptors are no longer interacting with one another (indicated by the gray region denoted ‘monomer’ in the plot in Figure 2). Error analyses were derived from the method described by Zhu and Hummer [30]. Specifically, errors were calculated based on the statistical variance of the raw data histograms estimated by block averages in increments of 200 ns. The dimerization constant, K_D_, and the free energy, ΔG, were calculated using an approach pioneered by Roux and colleagues [31], [32], specifically: equations (4) and (8) and a surface density for the POPC/cholesterol membrane patch of N_L_/A = 1.65×10^6^ µm^−2^, as recently described in the literature [22]. The free energy was calculated as ΔG = −RT ln (K_D_ × N_L_/A) at T = 300 K. Analyses of contact maps and energetic interactions were performed using Gromacs analysis tools [26].

## Results and Discussion

We have carried out umbrella sampling calculations for a total simulation time of ∼200 microseconds, to assess the relative stability of the three different crystallographic interfaces of opioid receptors that have been characterized to date. Specifically, we evaluated the strength of dimerization of MOPr and KOPr interfaces involving TM1, TM2, and H8, as well as the MOPr interface involving TM5 and TM6.

### Relative Stability of the Crystallographic Interfaces of MOPr

In the more compact TM5,6/TM5,6 crystallographic interface of MOPr, compared to that involving TM1, TM2, and H8, contacts are formed along the length of both of the TM5 and TM6 membrane spanning helices and the separation between the COMs of the helix bundles is ∼3.3 nm. The interface also comprises some involvement of the IL3, though this is somewhat cluttered by the inclusion of the T4L between residues R263 and E270 in each MOPr protomer, which contributes slightly to the interacting surface. The total buried solvent accessible surface area (SASA) in this interface between the MOPr protomers is 1606 Å^2^ (with 114 Å^2^ contributed by the T4L) compared to the 615 Å^2^ of the alternative TM1,2,H8/TM1,2,H8 crystallographic interface, which shows no involvement of the T4L, and a ∼4.0 nm separation between the COMs of the helical bundles [5].

**Figure 3:**
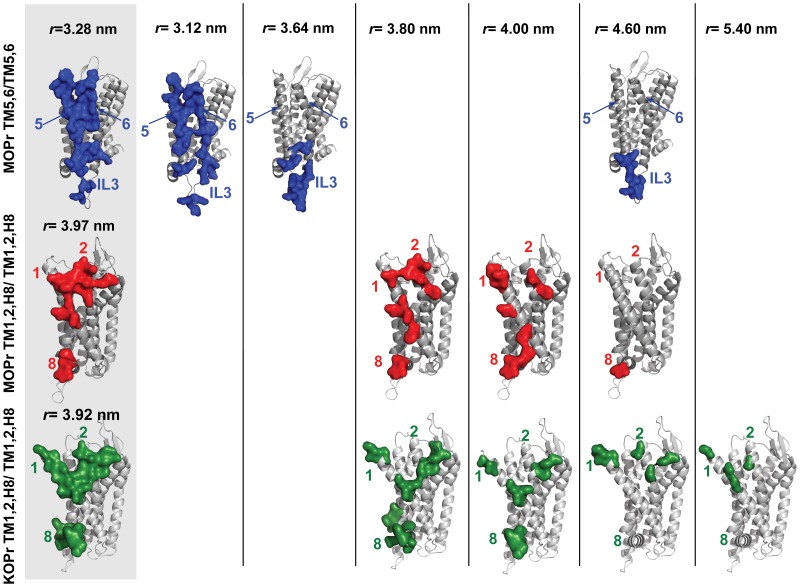
Contact surfaces (shown for one protomer in dimeric arrangements) at the values of r indicated by dashed grey lines in Figure 2. The crystal structure contact surfaces are shown (on grey background) and additional distances are r = 3.12 nm, r = 3.64 nm and r = 4.60 nm for TM5,6/TM5,6 MOPr interface, and r = 3.80 nm, r = 4.00 nm and r = 4.60 nm for MOPr and KOPr at TM1,2,H8/TM1,2,H8, with the addition of r = 5.40 nm for KOPr.

PMF free-energy curves for the two crystallographic interfaces of MOPr involving TM1, TM2, and H8, or TM5 and TM6 were derived from umbrella sampling simulations. These curves are shown in Figure 2 in red and blue colors, respectively. Inspection of these curves, as well as the values reported in Table 1, confirm a stronger free-energy of dimerization between receptors at the more compact crystallographic interface involving TM5 and TM6 than that involving TM1, TM2, and H8. In Figure 3, we have shown inter-protomer contact patterns for the minima from the PMF curves (identified in Figure 2 by dashed lines) and then at two large separation values (*r* = 4.60 nm and *r* = 5.40 nm). Contacts were defined as residues with an inter-protomer contact distance of less than 4.8 Å. The structures of the minima on which the contact maps are based were derived from clustering the frames of the simulation corresponding to each of the minima and taking the medoid of the largest cluster to be a representative structure.

For the TM5,6/TM5,6 interface of MOPr, close receptor packing leads to a densely populated interaction map involving residues spanning the entire TM5 and TM6 helices at short distances (*r*<3.64 nm). The corresponding PMF curve (blue line in Figure 2) shows a minimum at *r* = 3.12 nm, with shallower minima at *r* = 3.28 nm (corresponding to the crystal structure) and *r* = 3.64 nm. As shown in Table 2 and Figure 3, this contact interface is especially extensive at the minimum distance of *r* = 3.12 nm. Interactions span the full length of TM5 and TM6, as well as some of the residues comprising IL3 or EL2. The buried surface area in the coarse-grained minimum (at *r = *3.12 nm) is 16% of the entire monomeric surface. The corresponding value of buried surface area (i.e. T4L removed, and missing loop IL3 rebuilt) for the complete atomistic TM5,6/TM5,6 crystallographic dimer is 12%, and roughly equivalent to the SASA of the corresponding coarse-grained minimum in the PMF at r = 3.28 nm, as might be expected. Many of these close contacts are lost after the separation between the protomers approaches ∼3.64 nm, and beyond this, the direct inter-protomer interactions are dominated by residues in the intracellular ends of TM5, TM6 and the IL3. Five of the IL3 residues at this interface are not present in the crystallographic structure, having been replaced by the T4L insertion. This persistent interaction precludes a smooth plateau in the PMF free-energy of the TM5,6/TM5,6 interface at values of *r* larger than 3.64 nm, and prevents clear establishment of truly monomeric states for the receptors until well after this distance value. While the minimum of the PMF curve for MOP TM5,6/TM5,6 is deep, it is also narrow, which suggests that the packing of two protomers at this interface must be precise before a strong dimer would be formed under non-biased conditions. It must also be noted that while the elastic network used to maintain the secondary and tertiary structures is likely to impact the PMF, this effect would largely depend on the participation of loop regions at the interface, since the TM regions vary very little during simulation. The TM5,6/TM5,6 interface in MOPr contributes only ∼20% of both the SASA and the interaction energy between the protomers during the simulation of the lowest dimeric minimum, suggesting that the elastic network might influence approximately ∼20% of the corresponding PMF.

**Table 2:** Interacting residues at key values of separation (r) in the PMF (shown by dashed lines in Figure 2).

Interface	Crystal r = 3.28 nm	r = 3.12 nm	r = 3.64 nm		r = 4.60 nm	r = 5.40 nm
MOPr TM5,6/TM5,6	**226W EL2** **227Y EL2** **230N 5.36** **231L 5.37** **234I 5.40** **237F 5.43** **238I 5.44** **241F 5.47** **242I 5.48** **245V 5.51** **246L 5.52** ***249T 5.55*** **252Y 5.58** **253G 5.59** **255M 5.61** ***256I 5.62*** ***257L 5.63*** **259L IL3** **261S IL3** **263R IL3** **270E IL3** **279T 6.34** **280R 6.35** ***283L 6.38*** **290I 6.45** **294T 6.49** **298I 6.53** **301I 5.56** **302I 6.57** **304A 6.59** **305L 6.60** **306I EL3** **307T EL3**	170C 3.55*226W EL2 **230N 5.36** **231L 5.37** **234I 5.40** **237F 5.43** **238I 5.44** **242I 5.48** **245V 5.51** **246L 5.52** ***249T 5.55***250V 5.56***256I 5.62*** ***257L 5.63*** **263R IL3**266S IL3269K IL3276R 6.31277R 6.32**280R 6.35** ***283L 6.38***287A 6.42**290I 6.45**291V 6.46**294T 6.49** **298I 6.53** **301I 5.56** **302I 6.57** **305L 6.60** **306I EL2**	**252Y 5.58** ***256I 5.62*** ***257L 5.63***260K IL3261S IL3262V IL3**263R IL3**265L IL3266S IL3**270E IL3**271K IL3272D IL3276R 6.31***283L 6.38***		***256I 5.62*** ***257L 5.63*** **259L 5.65**260K IL3261S IL3**263R IL3**265L IL3**270E IL3**	–
**MOPr TM1,2,H8/TM1,2,H8**	**Crystal r = 3.97 nm**		**r = 3.80 nm**	**Minimum r = 4.00 nm**	**r = 4.60 nm**	**r = 5.40 nm**
	**69I 1.33** **72M 1.36** **76S 1.40** **77I 1.41** **80V 1.44** **122P 2.58** **125S 2.61** ***126V 2.62*** **129L 2.65** **130M 2.66** **135F EL1** **347F H8** **351C H8** **352I H8**		**69I 1.33** **76S 1.40** **77I 1.41** **80V 1.44**84F 1.48123F 2.59***126V 2.62*** **129L 2.65** **130M 2.66**348R H8**351C H8** **352I H8**	66V 1.30**69I 1.33**70T 1.34**77I 1.41**84F 1.48123F 2.59***126V 2.62***350F H8**351C H8** **352I H8**	**352I H8**	–
**KOPr TM1,2,H8/TM1,2,H8**	**Crystal r = 3.92 nm**		**r = 3.80 nm**	**r = 4.00 nm**	**r = 4.60 nm**	**r = 5.40 nm**
	***56P 1.29*** ***59P 1.32*** **60V 1.33** **63T 1.36** **64A 1.37** **66Y 1.39** **67S 1.40** **68V 1.41** ***70F 1.43*** **71V 1.44** **113P 2.58** **114F 2.59** **116S 2.61** **117T 2.62** **120L 2.65** **125P EL1** **126F EL1** **331A 7.54** **332F 7.55** **338K H8** **341F H8** **342R H8** **346F H8** **347P H8**		***56P 1.29***57A 1.30***59P 1.32*** **67S 1.40** **68V 1.41** ***70F 1.43*** **71V 1.44** **113P 2.58** **114F 2.59** **124W EL1** **126F EL1** **328I 7.51** **332F 7.55** **341F H8** **342R H8**343D H8**346F H8**	57A 1.30**60V 1.33** **67S 1.40** ***70F 1.43*** **71V 1.44** **114F 2.59** **126F EL1** **341F H8**343D H8345C H8**346F H8**347P H8	55S 1.28***56P 1.29***57A 1.30***59P 1.32*** **113P 2.58**120L 2.65**126F EL1**	***56P 1.29*** **63T 1.36** **67S 1.40** **116S 2.61**

Cut-off value is shortest inter-protomer contact distance less than 4.8 Å between any atoms, for representative structures from the different simulation windows indicated by the dashed lines. Bolded residues are those present in the crystallographic KOPr and MOPr structures at the same cut-off values. *The interaction with this residue comes from the palmitoyl chain of the C3.55, which is not present in the crystallographic structures. Italicized residues are mentioned specifically in the text.

Although the large number of contacting residues in the TM5,6/TM5,6 interface between protomers of the MOPr makes it significantly more stable than the TM1,2,H8/TM1,2,H8 crystallographic interface of the MOPr, the analyses herein cannot confirm that either interface would form spontaneously by self-association in an explicit membrane environment. If the TM5,6/TM5,6 interface did form spontaneously, analysis of the contacts formed at the largest separation value before interface disruption (see Table 2 column corresponding to *r* = 4.60) suggests residues that might contribute more significantly than others to its stability, and that might be worthy of experimental testing. Notably, although the engineered palmitoyl chain at position C3.55 of one of the two MOPr protomers appears to form contacts with residues I238^5.44^ and I242^5.48^ of the adjacent protomer at the representative dimeric minimum of the TM5,6/TM5,6 interface, these contacts are not retained at values of *r* beyond the minimum state, thus suggesting only a minimal contribution of the palmitoyl chain to the stability of the TM5,6/TM5,6 interface. Some trapping of cholesterol against TM4 by the palmitoylated C3.55 residue, recently suggested by a homology model of the MOPr [24], is observed in some of the windows of the simulations discussed herein, but the molecule is not located close enough to directly affect the TM5,6/TM5,6 interface.

### Relative Stability of the Crystallographic Interfaces of MOPr and KOPr Involving TM1, TM2, and H8

The TM1,2,H8/TM1,2,H8 crystallographic interfaces of MOPr and KOPr are very similar interfaces with a separation between the COMs of the two protomers measured to be ∼4 nm in both cases. However, they are not identical. The most prominent difference between these interfaces stems from the conformation of TM1, being curved slightly backward toward the TM7 of the bundle in MOPr, but very straight, and protruding outwards from the rest of the bundle, in KOPr. As a consequence, there is a larger buried SASA at this interface in KOPr (1,100 Å^2^ ) [6] compared to MOPr (615 Å^2^) [5], and a slightly different arrangement of the protomers relative to one another. In the KOPr, the principal axes of each of the two protomers comprising the dimer are parallel to one another, meaning that both protomers are expected to sit perpendicular to the membrane environment. For MOPr, the principal axes of each of the two protomers at the TM1,2,H8/TM1,2,H8 interface are offset by a torsion angle of approximately 20°, which may give rise to local differences in the membrane to accommodate each protomer.

Not surprisingly, inter-protomer interactions at the TM1,2,H8/TM1,2,H8 interface are different for the two receptor subtypes, with KOPr exhibiting a) a more significant degree of contact at the extracellular terminus of TM1, which interacts directly with TM2 and EL1 on the adjacent protomer, and b) more significant inter-protomer contacts for H8, than observed in MOPr (see Table 2). The observed differences in the crystallographic contact map also render it unsurprising that, despite ostensibly involving the same helices, the two crystallographic interfaces of MOPr and KOPr exhibit very different PMF curves, in terms of both shape and depth (see Figure 2). This results in significantly different relative free-energies of dimerization (see Table 1). The PMF curve for MOPr has two minima, at approximately the same depth, with values of *r = *3.80 and 4.00 nm and similar contact map profiles. The PMF curve is very much deeper for KOPr (shown in green) than for MOPr (shown in red), and the estimated free-energy of the interface suggests that the KOPr TM1,2,H8/TM1,2,H8 interface is very much more stable than for MOPr. If we examine the contacts between receptors at larger separations, as reported in Table 2, there are significant contacts for KOPr at r = 5.40 nm. These interactions involve the extracellular ends of TM1 and TM2, and they continue to contribute to the free-energy of dimerization at this interface, even at large protomer-protomer separation. In contrast, the most persistent inter-protomeric contact at the TM1,2,H8/TM1,2,H8 interface of MOPr is seen at *r* = 4.60 nm and is the terminal residue, I352, in H8. The paucity of inter-protomer interactions at this separation corresponds with the abrupt plateau in the shape of the PMF curve for the MOPr TM1,2,H8/TM1,2,H8 interface beyond *r* = 4.50 nm. Taken together, these observations strongly suggest that the shape of the TM1,2,H8/TM1,2,H8 interface, particularly the position of TM1, either pointing away from the rest of the helical bundle as in KOPr, or toward the helical bundle, as in MOPr, influences the shape of the PMF describing the dimerization between receptors. However, since it is known that TM1 can adopt more than one conformation even within the same crystal lattice, as seen in the case of turkey β1-adrenergic receptor [33], we cannot rule out the possibility of changes in the conformation of TM1 of KOPr. The elastic network, used to maintain the secondary and tertiary structure of the receptors under the CG force field during the simulations presented herein, prevents sampling of these different conformations. If the TM1 adopts the most extended conformation, for either receptor subtype, during unbiased association between protomers, we speculate that the most stable receptor dimers would be formed.

Sequence comparison between the mouse MOPr and human KOPr also indicates some variation in the regions of TM1 and TM2 that may contribute to the differential stability of the TM1,2,H8/TM1,2,H8 interface for the two opioid receptor subtypes. Specifically, in the crystallographic structures and in the dimeric minima of KOPr and MOPr, variations in the contacts correspond to: a) different residues at positions 1.29 (a methionine in MOPr and a proline in KOPr), 1.32 (an alanine in MOPr and a proline in KOPr), 1.36 (a methionine in MOPr and a threonine in KOPr) and 2.62 (a valine in MOPr and a threonine in KOPr); b) a contact at position 1.43 for KOPr (phenylalanine), but not for MOPr (cysteine); and c) a contact at position 1.48 for MOPr (phenylalanine) but not for KOPr (valine). Notably, in a monomeric state, residues 1.32, 1.36 and 2.62 would be interacting directly with the lipid membrane, a potentially unfavorable energetic state, which, in this case, can be alleviated by receptor interaction at the KOPr TM1,2,H8/TM1,2,H8 interface. In MOPr, 1.32 is less exposed to the lipid membrane than in KOPr, owing to the bent conformation of TM1 in the MOPr crystal structure. Of these residues, P1.29 at the extreme of TM1 is among the residues remaining in contact up to the largest separation (*r* = 5.40 nm) for KOPr, and T1.36 is in contact in both the crystal structures, and at the largest separation, but does not appear consistently in the contact maps at the intermediate distances.

## Conclusions

In this study, we have presented the calculation of the potential of mean force for the dimerization between protomers of KOPr or MOPr, at interfaces inferred from recent crystal structures of the receptors. Our results indicate that each of the crystallographic interfaces we have investigated forms an energetically viable dimeric configuration in an explicit membrane environment, with the KOPr TM1/TM/H8 interface being the most stable of all, followed by MOPr TM5,6/TM5,6, and lastly by MOPr TM1,2,H8/TM1,2,H8. Thus, dimerization between opioid receptors appears to vary widely among receptor subtypes, even at interfaces involving the same helices, and we discuss the contributions from the receptor sequence and shape to the relative interface stability. Although further experiments are still necessary to assess the likelihood of a specific opioid receptor region forming part of a physiologically relevant dimerization interface, our studies represent the first step in this direction by providing testable hypotheses of residues that, if mutated, could advance knowledge of the role of dimerization in opioid receptor function.
